# Incidence and location of heterotopic ossification following hip arthroscopy

**DOI:** 10.1186/s12891-020-3150-7

**Published:** 2020-02-28

**Authors:** Long Zheng, Jung-Mo Hwang, Deuk-Soo Hwang, Chan Kang, Jeong-Kil Lee, Young-Cheol Park

**Affiliations:** 1 Department of Orthopedic Surgery, Yanbian University Hospital, Yanji, China; 2 Department of Orthopedic Surgery, Chungnam National University Hospital, 266, Munwha-ro, Jung-gu, Daejeon, 35015 South Korea

**Keywords:** Hip arthroscopy, Heterotopic ossification, Portal, Capsulotomy

## Abstract

**Background:**

We investigated the incidence and location of heterotopic ossification (HO) following hip arthroscopy.

**Methods:**

This retrospective study enrolled 327 patients who underwent hip arthroscopy from January 2010 to December 2015. From this cohort, we extracted an HO group with simple radiographs or three-dimensional computed tomography (3D CT). Findings consistent with HO were classified according to the Brooker classification aided with 3D CT for the location of HO. The indication for revision arthroscopic excision of HO was painful, functional impairment of the hip. Patient clinical outcomes were assessed pre- and postoperatively, with modified Harris Hip Scores (mHHS), a visual analogue scale (VAS) for pain, and the Hip Outcome Score-Activity of Daily Living (HOS-ADL) and Sport Specific Subscales (HOS-SSS).

**Results:**

In all, 14 (4.28%) of the 327 patients had confirmed HO radiographically. The mean follow-up was 39 months. In 13 patients, HO formed in the central area of the arthroscopic portals or capsulotomy. Ten patients had Brooker Grade 1 and four had Grade 2. At the last follow-up, 12 asymptomatic patients had significant (*P* < 0.001) improvements in all clinical outcome scores (mHHS, pain VAS, HOS-ADL, and HOS-SSS). Two patients developed symptoms sufficient to require revision hip arthroscopy for HO excision. After revision hip arthroscopy, both symptomatic patients had improved significantly in all clinical outcomes at the final follow-up.

**Conclusions:**

HO is a minor complication of hip arthroscopy, but sometimes induces severe pain and functional impairment. Usually, HO forms in the arthroscopic portal or capsulotomy area.

## Background

Heterotopic ossification (HO) after hip arthroscopy is the abnormal formation of mature lamellar bone within the extra-skeletal soft tissues, usually between the muscle and joint capsule and is a minor complication after hip arthroscopy [1–3]. Its exact etiology is unknown, although several factors have been proposed as potential mediators, including altered prostaglandin production, hormone activity, and tissue oxygen and calcium levels, and prolonged immobilization [4]. The reported prevalence of HO following hip arthroscopy ranges from 0 to 11.5% [2, 3, 5–7]. Sometimes, HO after hip arthroscopy may produce functional impairment, pain, impingement, and decreased range of motion [1]. Historical methods to reduce HO following hip surgery have involved postoperative radiation or chemoprophylaxis with nonsteroidal anti-inflammatory drugs (NSAIDs) [5]. Despite effective chemoprophylaxis, HO still occurs in a small percentage of patients after hip arthroscopy [8]. Although HO is frequently recognized radiographically in the postoperative period, it is unclear whether HO is relevant clinically [8]. The clinical consequences of HO are debatable, and little is known about the prognosis of symptomatic HO after hip arthroscopy. In this study, we retrospectively reviewed consecutive cases of HO after hip arthroscopy to determine the incidence and location of HO following hip arthroscopy and to find ways to reduce HO.

## Methods

### Patient selection and clinical evaluation

This study was approved by the institutional review board of Chungnam National University School of Medicine (CNUH 2018–05–049). All cases that underwent hip arthroscopy at our institution from January 2010 to December 2015 were reviewed retrospectively. Only patients with complete medical records that included operation records and follow-up scoring and preoperative, postoperative, and follow-up radiographs were included. Patients were excluded if they had previous hip conditions, such as previous hip surgery, Legg–Calves–Perthes disease, diffuse idiopathic skeletal hyperostosis, ankylosing spondylitis, avascular necrosis, and dysplasia. There were 327 patients (101 females, 226 males) with a mean age of 36.3 ± 12.9 (range 14–69) years. The indications for arthroscopic excision of HO were symptoms sufficient to cause pain and functional impairment of the hip that differed from the patient’s preoperative labral or intra-articular symptoms. Patients were evaluated at 2 weeks, 3 and 6 months, and then annually after surgery or more frequently if necessary. At each visit, we obtained the modified Harris Hip Scores (mHHS) and Hip Outcome Score-Activity of Daily Living (HOS-ADL) and Hip Outcome Score-Sport Specific Subscales (HOS-SSS). To estimate pain intensity, patients were asked to rate their pain on a visual analogue scale (VAS). Three reviewers evaluated all radiographs to identify patients in whom HO had developed. Findings consistent with HO were classified according to the Brooker classification [9] and three-dimensional computed tomography (3D CT) was used to locate the HO.

### Statistical analysis

All statistical analyses were performed using IBM SPSS Statistics (ver. 24.0; IBM, Armonk, NY, USA). Differences between the preoperative and latest follow-up clinical outcomes were compared using the paired *t*-test. In the two patients who underwent revision hip arthroscopy, statistical analyses were not performed. Differences were considered statistically significant if *P <* 0.05.

### Surgical method and post-operative management

All arthroscopic procedures were performed by the senior surgeon (DSH) while the patients were in the supine position on a traction table under general anesthesia. The procedure usually started in the central compartment. Traction was applied and a guidewire inserted into the joint through an anterolateral portal using a puncture needle and C-arm image intensifier control. The portal was dilated and a 70° scope was inserted through the portal. A mid-anterior portal was established under direct visualization outside-in. Sometimes we made additional portals. After the portal setup, an interportal capsulotomy was performed between the mid-anterior and anterolateral portals for visualization and instrument access. The central and peripheral compartments were evaluated systematically and the results recorded. Arthroscopic labral repair, partial debridement, or reconstruction was performed for a damaged labrum. Additional procedures performed include acetabuloplasty, femoroplasty, loose body removal, microfracture, ligamentum teres shrinkage, partial iliopsoas tenotomy, and percutaneous screw fixation. Bony debris from the osteochondroplasty was removed by suction and irrigation. Capsular closure/plication was not performed in any patient.

The postoperative rehabilitation was similar for most patients, except those with microfractures who were prescribed no weight-bearing for 6 weeks. From the first postoperative day, stationary cycling, partial weight-bearing ambulation, and pendulum exercises were encouraged to prevent soft tissue adhesions and promote early recovery. We also prescribed prophylactic postoperative NSAIDs for 3 to 6 weeks in all patients.

For patients with symptomatic HO after conservative treatment, arthroscopic excision was performed. Revision hip arthroscopy was performed by the same senior surgeon while the patients were in the supine position on a traction table under general anesthesia. Routine diagnostic arthroscopy of the central and peripheral compartments was performed with the standard two-portal technique using the anterolateral and mid-anterior portals. The position of the HO was identified using a C-arm intensifier, and then radiofrequency and a shaver were used to desquamate the ossification from the soft tissue. Finally, a grasper was used to remove the HO. A large HO was osteotomized using a burr to allow removal through the arthroscopic portals.

All patients followed a standard post-revision protocol. The patients were allowed weight-bearing ambulation and pendulum exercises of the hip from the day of surgery. The next day, radiotherapy (10 Gy) and indomethacin (100 mg/day for 6 weeks) were commenced. The patient was released 1 week postoperatively when the radiotherapy was completed.

## Results

In all, 14 (4.28%; 1 female, 13 males) of the 327 patients had HO confirmed radiographically; their mean age was 34.7 ± 12.0 (range 19–57) years. The mean follow-up was 39.4 ± 12.4 (range 24–80) months. The leading intraoperative diagnosis was femoroacetabular impingement (FAI), found in 10 patients (7 cam type, 3 mixed), followed by synovial chondromatosis (2 patients), internal snapping hip (1 patient), and acetabular posterior wall fracture (1 patient). In 13 patients (92.9%), the HO formed in the central area of the arthroscopic portals (mid-anterior or anterolateral portion of the hip joint) or capsulotomy area during the primary surgery. Ten patients had Brooker Grade 1 HO and four had Grade 2 HO (Table 1).

**Table 1:** Characteristics of the 14 heterotopic ossification patients

Patient number	Sex	Age (years)	Primary diagnosis	Primary AS	Brooker grade	Location	Revision AS
1	M	19	Mixed FAI	Labral repair, Acetabulofemoroplasty	2	Anterolateral	HO excision, Indomethacin, Radiotherapy
2	F	32	Mixed FAI	Labral repair, Acetabulofemoroplasty	1	Anterolateral	None
3	M	27	Cam FAI	Labral repair, Femoroplasty	2	Anterior & anterolateral	None
4	M	48	Cam FAI	Labral repair, Femoroplasty	1	Anterolateral	None
5	M	41	Cam FAI	Labral repair, Femoroplasty	1	Anterolateral	None
6	M	24	Internal snapping hip	Iliopsoas tendon relese	2	Anterior	HO excision, Indomethacin, Radiotherapy
7	M	46	Cam FAI	Partial labrectomy, Femoroplasty	1	Anterolateral	None
8	M	32	Synovial chondromatosis	LB removal, Synovectomy	1	Anterolateral	None
9	M	22	Synovial chondromatosis	LB removal, Synovectomy	2	Anterior	None
10	M	42	Mixed FAI	Labral repair, Acetabulofemoroplasty	1	Anterolateral	None
11	M	57	Acetabular posterior wall fracture	LB removal, AS reduction & screw fixation	1	Posterior	None
12	M	25	Cam FAI	Labral repair, Femoroplasty	1	Anterior	None
13	M	29	Cam FAI	Partial labrectomy, Femoroplasty	1	Anterolateral	None
14	M	48	Cam FAI	Labral repair, Femoroplasty	1	Anterior	None

*HO* Heterotopic ossification, *AS* Arthroscopy, *FAI* Femoroacetabular impingement, *LB* Loose body

Twelve asymptomatic HO patients showed significant improvement in all clinical outcomes (all *P* < 0.001). At the final follow-up, the mHHS, HOS-ADL, HOS-SSS, and pain VAS significantly improved from 61.9, 59.4, 53.3, and 6.9, respectively, to 89.5, 83.7, 84.3, and 1.9 (Table 2). There were no intra- or perioperative complications. All patients returned to their preoperative level of function.

**Table 2:** Clinical outcomes in asymptomatic heterotopic ossification patients (mean ± SD)

Outcomes measure	Preoperative	Latest follow-up	*P* values
mHHS	61.9 ± 6.4	89.5 ± 9.7	< 0.001
HOS-ADL	59.4 ± 8.9	83.7 ± 11.4	< 0.001
HOS-SSS	53.3 ± 7.7	84.3 ± 12.3	< 0.001
Pain VAS	6.9 ± 0.7	1.9 ± 1.4	< 0.001

*SD* Standard deviation, *mHHS* Modified harris hip scores, *HOS-ADL* Hip outcome score-activity of daily living, *HOS-SSS* Hip outcome score-sport specific subscales, *VAS* Visual analogue scale

**Fig. 1.**
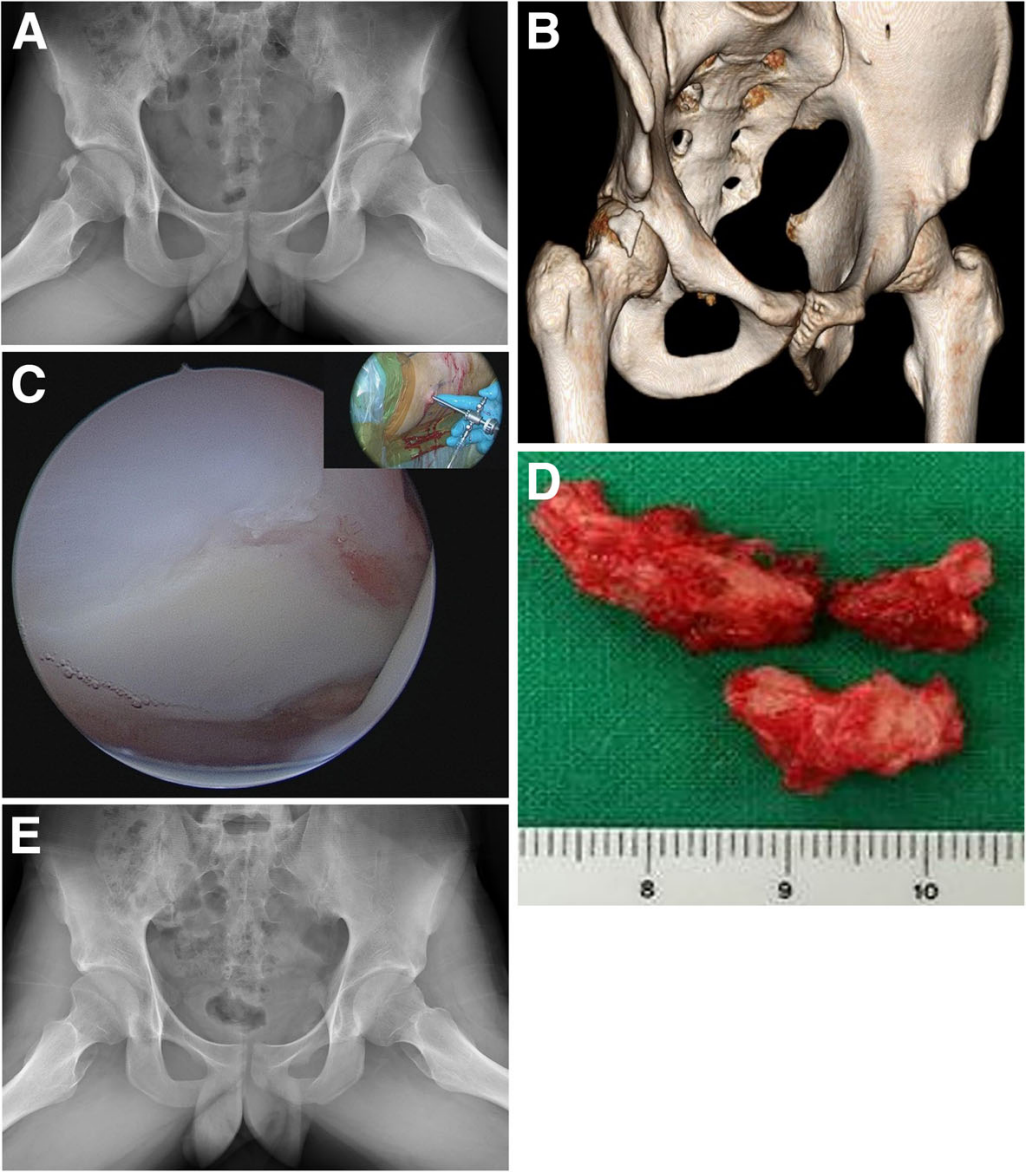
**a**, **b** Frog-leg lateral radiograph and 3D CT scan of a 19-year-old male patient, acquired 1 year after arthroscopic labral repair and acetabulofemoroplasty. Heterotopic ossification was classified as Brooker grade 2, with the largest measurement (width) being 21 mm. **c**, **d** Heterotopic ossification was located in anterolateral joint capsule and removed with arthroscopy. **e** Frog-leg lateral radiograph showed no evidence of heterotopic ossification at 2 year follow-up

Two patients (0.61%) developed pain and functional impairment that differed from the preoperative labral or intra-articular symptoms, so we performed revision hip arthroscopy for HO excision: one patient had FAI and the HO was located in the anterolateral joint capsule (Fig. 1) and the other patient had an internal snapping hip and the HO was located in the iliopsoas muscle. Both of these patients had Brooker grade 2 HO and there was no other pathology around the hip (Table 1). Before revision, the mHHS was 52/55, HOS-ADL 58/51, HOS-SSS 56/49, and pain VAS 7/7. Following revision hip arthroscopy for HO excision, each score had improved with mHHS 92/85, HOS-ADL 93/85, HOS-SSS 91/87, and pain VAS 0/1 (Table 3). Over their follow-up, both patients who underwent revision hip arthroscopy improved gradually and ultimately returned to their levels of recreational athletics before surgery. There was no evidence of HO on the patients’ 2-year follow-up radiographs.

**Table 3:** Clinical outcomes after arthroscopic excision of symptomatic heterotopic ossification

Scoring reporting	mHHS		HOS-ADL		HOS-SSS		Pain VAS	
	Case							
	No. 1: FAI	No. 2: ISH	No. 1	No. 2	No. 1	No. 2	No. 1	No. 2
Prior to revision	52	55	58	51	56	49	7	7
Lastest post-revision arthroscopy follow-up	92	85	93	85	91	87	0	1

*mHHS* Modified harris hip scores, *HOS-ADL* Hip outcome score-activity of daily living, *HOS-SSS* Hip outcome score-sport specific subscales, *VAS* Visual analogue scale, *FAI* Femoroacetabular impingement, *ISH* Internal snapping hip

## Discussion

Characteristics that may predispose patients to HO following hip arthroscopy include capsulotomy and acetabuloplasty with osteochondroplasty for mixed-type FAI and a large amount of bone resection, although these arthroscopic procedures have not been specifically studied [3, 10]. Similarly, the proximity of periosteal disruption to damaged musculature about the hip may contribute to a high propensity for HO after other hip surgery, including hip arthroplasty, acetabular trauma, and surgical hip dislocation [11, 12]. Rath et al. [13] hypothesized that capsule repair after hip arthroscopy decreased HO by blocking the interface between the injured periosteum and necrotic or damaged muscle, while Amar et al. [14] reported that capsule repair did not seem to alter the rate of HO compared to a control group of patients in whom the capsulotomy was not repaired. In our study, most patients underwent capsulotomy or osteochondroplasty, but the incidence of HO was not as high (4.28%) as reported. In 13 of the 14 patients (92.9%), HO formation was in the central area of the arthroscopic portals placed during the primary surgery (anterior or anterolateral portion of hip joint), so we thought that gentle soft tissue handling (minimizing portal trauma and peri-articular soft tissue damage) and fluid evacuation of the bony debris from the osteochondroplasty are more important than the capsulotomy or osteochondroplasty for preventing HO after hip arthroscopy; however, we did not have sufficient patients to assess the power of this comparison, which merits further study.

Symptoms of HO include articular disability, stiffness, pain, and crepitation, but most have minimal or no clinical or functional significance. Moreover, in around 64% of patients the ectopic ossification is a self-limited naturally resolving entity that does not require surgical intervention [15]. Nevertheless, if persistent pain or limited articular activity is seen, then it is important to suspect HO, although the clinical manifestations are difficult to isolate from other sources of postoperative pain. A mechanical blockage can explain the motion pain if the HO has formed in the plane of motion, mainly anterior and lateral. In our revision hip arthroscopy for HO excision patients, the HO was located anterior to the iliopsoas in one and anterolateral to the capsule in the other. Examinations showed that severe pain was induced during hip flexion, adduction, and internal rotation. This motion pain had resolved nearly completely at 1 year and no further treatment was required at the most recent follow-up.

HO lesions must be allowed to mature fully before surgical excision. Bedi et al. [5] treated 7 of 29 patients who developed HO postoperatively with revision surgery to excise the HO a mean of 11.6 months after the prior hip arthroscopy. Beckman et al. [10] reported arthroscopic HO resection in 9 of 34 patients who developed HO at 12 months postoperatively. This 12-month period was used to ensure full maturation of the HO and to allow for adequate recovery from the prior hip arthroscopy. Animal studies show that ectopic bone formation begins within 5 days of injury [16]. In humans, ossification is evident radiographically by 6 weeks and does not progress further at 12 to 24 weeks [17, 18]. Therefore, we treated both patients who developed symptomatic HO postoperatively with revision hip arthroscopy 12 months after the prior hip arthroscopy.

The best treatment of HO is prevention. Low-dose irradiation and NSAIDs are two common methods to prevent HO. NSAIDs are thought to limit HO by inhibiting cyclooxygenase and preventing prostaglandin synthesis. This may result in the inhibition of mesenchymal cell proliferation [19] and differentiation of mesenchymal cells into osteogenic cells [20]. Various NSAIDs have been shown to decrease postoperative HO [21, 22]. Indomethacin is the most extensively studied NSAID for use in preventing HO. Bedi et al. [5] found that taking indomethacin after hip arthroscopy is effective at preventing HO, particularly in males after osteoplasty. They found that HO developed postoperatively in 29 (21 males, 8 females) of 616 hip procedures (4.7%). The HO rate for cases with and without prophylactic indomethacin was 1.8% (6 of 339) and 8.3% (23 of 277), respectively. The duration of indomethacin prophylaxis is generally 6 weeks, although some reports suggest 3 weeks is effective [20]. Ionizing radiation influences rapidly dividing cells by altering the nuclear DNA. Thus, early postoperative radiation may prevent the differentiation of some pluripotent mesenchymal cells into osteoblasts [23]. There is currently little evidence to support the routine use of prophylaxis for HO in arthroplasty patients, but some investigators recommend prophylaxis for high-risk patients [24]. In our study, all of the patients were treated with an NSAID (range 3–6 weeks) after primary hip arthroscopy and underwent prophylactic radiotherapy (10 Gy) and were prescribed indomethacin (100 mg/day, for 6 weeks) after revision hip arthroscopy.

There were several limitations to this study. First, it was a single-institution retrospective study, so the number of patients was too small to generate significant results. Second, the radiological evaluation of developing HO is subjective. To increase the objectivity, one radiologist and two orthopedic surgeons, all board certified, evaluated the radiological imaging. Third, this study did not make comparisons with a non-prophylaxis group. Fourth, although one senior surgeon performed the hip arthroscopy, some cases underwent more aggressive osteochondroplasty or a more extended capsulotomy, which resulted in more soft tissue damage and bony debris. Further well-controlled prospective studies should be conducted to address different arthroscopic techniques to reduce HO after hip arthroscopy.

## Conclusions

Heterotopic ossification is a minor complication of hip arthroscopy and has minimal or no clinical and functional significance in most patients. Usually, HO is located in the arthroscopic portal or capsulotomy area. Arthroscopic removal of the HO is indicated in patients with severe pain or limitation in daily activities after conservative treatment. Preventing HO formation after hip arthroscopy requires careful attention to gentle soft tissue handling and suction and irrigation to remove all bony debris from the osteochondroplasty.

## Data Availability

The datasets used and/or analyzed during the current study are available from the corresponding author on reasonable request.

## Abbreviations

3D CT: Three-dimensional computed tomography (3D CT)
FAI: Femoroacetabular impingement
HO: Heterotopic ossification
HOS-ADL: Hip Outcome Score-Activity of Daily Living
HOS-SSS: Hip Outcome Score-Sport Specific Subscales
mHHS: Modified Harris Hip Scores
NSAID: Nonsteroidal anti-inflammatory drug
VAS: Visual analogue scale
